# *Per os* colchicine administration in cholesterol fed rabbits: Triglycerides lowering effects without affecting atherosclerosis progress

**DOI:** 10.1186/s12944-017-0573-8

**Published:** 2017-09-26

**Authors:** Vaios Vasileios Kaminiotis, George Agrogiannis, Panagiotis Konstantopoulos, Vasiliki Androutsopoulou, Laskarina Maria Korou, Ioannis S. Vlachos, Ismene A. Dontas, Despina Perrea, Dimitrios C. Iliopoulos

**Affiliations:** 1 Laboratory for Experimental Surgery and Surgical Research, Medical School, National and Kapodistrian University of Athens, Agiou Thoma 15B, Goudi, 115 27 Athens, Greece; 2 First Department of Pathology, National and Kapodistrian University of Athens School of Medicine, Agiou Thoma 17, Goudi, 115 27 Athens, Greece; 3 Laboratory for Research of the Musculoskeletal System “Th. Garofalides”, School of Medicine, National and Kapodistrian University of Athens School of Medicine, Nikis 2, Kifissia, 145 61 Athens, Greece

**Keywords:** Colchicine, Rabbits, Atherosclerosis, Triglerycide, IL-18, Leptin, Insulin

## Abstract

**Background:**

Atherosclerosis is a chronic inflammatory disease that is promoted, among others, by pro-inflammatory cytokines such as IL-1β and IL-18 produced by NLRP 3 inflammasome. Development of atherosclerotic lesions is also affected by leptin. Furthermore, inflammasome’s action is interfered with other inflammatory diseases, like diabetes. On the other hand, colchicine is reported to act as anti-inflammatory agent inhibiting inflammasome’s action and stabilizing atherosclerotic lesions. The purpose of this study is to investigate the effect of per os colchicine on the de novo formation of atherosclerotic lesions and on the levels of IL-18, leptin and insulin in cholesterol-fed rabbits.

**Methods:**

Twenty-three male, 2 months old New Zealand White rabbits, were seperated in 3 groups and were fed with different types of diet for 7 weeks: standard, cholesterol 1% *w*/w and cholesterol 1% w/w plus colchicine 2 mg/kg body weight. Blood was collected for biochemical measurements and conduction of ELISA for leptin, IL-18 and insulin. Histologic examination of stained with eosin and hematoxylin aorta specimens was performed. Aortic intimal thickness was evaluated using image analysis. The statistical analysis included non-parametric tests: a) paired-sample Wilcoxon test, b) Spearman correlation coefficient and c) Kruscal-Wallis test.

**Results:**

Triglerycide levels were decreased in cholesterol plus colchicine group in the end of the experiment (*p* < 0.05), whereas the cholesterol group had increased levels. No statistical differences were observed in the levels of IL-18, leptin and insulin between groups. Likewise, there was neither any correlation between IL-18, leptin and intima thickness nor between IL-18 and glucose and between leptin and weight. In cholesterol and colchicine group there was a strong positive correlation between IL-18 and insulin levels in the 4th week (*r*_s_ = .66, *n* = 10, *p* < 0.05), whereas in the 7th week this correlation became strong negative (*r*_s_ = −.86, *n* = 10, *p* < 0.05). Finally, intima thickness in the ascending and thoracic aorta of the cholesterol and colchicine group was significantly greater than that of the other groups (*p* < 0.05).

**Conclusions:**

Per os administration of colchicine did not influence atherosclerosis progression in cholesterol-fed rabbits, levels of IL-18, insulin and leptin. We encountered the attenuating role of colchicine on TG levels.

## Background

Atherosclerosis is related with major adverse cardiac events such as myocardial infarction and stroke, the two leading causes of death in the world [1]. Being a low-grade inflammatory process, atherosclerosis emerges as an attractive target for anti-inflammatory drugs [2, 3]. These drugs could regulate the inflammatory pathway that converts a stable to a vulnerable plaque, causes thrombosis and increases the risks for severe clinical events.

Current evidence has shown that NOD-like receptor protein 3(NLRP 3) inflammasome – a protein complex of the cytosol, that is activated by pathogen or host signals, such as cholesterol crystals [4–7] – promotes the secretion of interleukin (IL)-1β and IL-18. It is known that these inteleukins contribute to the progression and instability of atherosclerotic plaques [8, 9]. Inflammasome’s action is considered to be responsible for the development of various disorders including inflammatory, autoimmune and metabolic diseases, such as type 2 diabetes (T2D) [10, 11]. It has been reported that IL-18 levels are associated with the evolution of diabetes. IL-18 induced mice macrophages in pancreatic islets to produce nitric oxide and cytokines that eventually led to apoptotic death in transplanted islets [12]. In non-obese diabetic (NOD) mice, pancreatic cells may express high levels of IL-18 which causes insulitis [13].

Moreover, recent studies showed that leptin affects the development of atherosclerosis. Subphysiological to physiological levels of leptin in leptin-deficient low-density lipoprotein receptor (LDLR) knockout (*LDLR* ^*−/−*^ *;ob/ob*) mice seem to reduce the atherosclerotic lesions in an indirect way, whereas the opposite effect takes place with higher doses of leptin [14]. Apart from that, reduced inflammatory response and reduced cholesterol accumulation in macrophages in *ob/ob* mice was detected, proposing leptin as a key mediator in the formation of foam cells [15].

Colchicine, a well-known anti-inflammatory drug, exerts its actions through binding to free tubulin dimers and therefore disrupting microtubule polymerization [16]. Lately, several studies were conducted for the possible preventive role of colchicine in atherosclerosis and its adverse effects. The LoDoCo trial showed that 3 years administration of low dosage of colchicine in patients with stable coronary disease reduced cardiovascular events [17]. It was also demonstrated that short-term administration of colchicine in patients with acute coronary syndrome (ACS) reduced the production of IL-1β and IL-18 in coronary vessels [18]. It has been recently reported that colchicine exerts one of its anti-inflammatory properties through inhibition of NLRP3 inflammasome formation in macrophages [19] as well as scaling down neutrophil infiltration [20].

Rabbit is the most common animal model studied in experimental research on atherosclerosis. Dietary interventions in these animals are extensively used investigating the beneficial action of hypolipidemic and anti-inflammatory agents in atherosclerotic processes. The induction of atherosclerotic lesions in rabbits is easily achieved after feeding the animals a high-cholesterol diet for a period of 8–12, even 7 weeks, depending on the concentration of cholesterol added in the standard chow [21–24]. Since studies have shown that cell proliferation in thoracic and abdominal aortas as well as fatty streaks begin early after the administration of hypercholesterolemic diet in rabbits [25, 26] and the events that trigger atherosclerosis resemble to those of humans [27, 28], we considered the rabbit as the most suitable animal model.

Taking into account the anti-inflammatory action of colchicine and its role in stabilizing atherosclerotic lesions, we hypothesized that per os administration of colchicine in a hypercholesterolemic rabbit model could inhibit the formation of atherosclerotic lesions. To our knowledge, the effect of per os administration of colchicine on the formation of atherosclerotic plaques has not been investigated to date. Apart from that, we investigated the effect of colchicine on the levels of inflammatory and metabolic indices, such as IL-18, leptin and insulin during prolonged hypercholesterolemia in New Zealand White rabbits. If our hypothesis could be confirmed, then a new drug could be added in the prevention of cardiovascular disease and the role of orally administered anti-inflammatory drugs in atherosclerosis could be reviewed from a different perspective.

## Methods

### Animal models and experimental design

Twenty-three male, 2 months old, New Zealand White rabbits (*Oryctolagus cuniculus*) (body weight 3.51 ± 0.19 kg, mean ± SD) were individually housed in stainless steel wire-bottom cages. A farm breeding rabbits for experimental purposes in the Attica region provided the animals. The rabbits were housed one per cage and were kept in a temperature-controlled environment (19 ± 1 °C with 50 ± 5% relative humidity) with a 12-h light/dark cycle (5:30 am to 5:30 pm) in an air-conditioned room with 15 air changes/h and had free access to food and tap water. During their stay the everyday consumption of tap water was measured individually. A veterinarian examined the animals clinically during the entire experimental period. All possible steps were taken to avoid animal suffering at each stage of the experiment. The interventions that could cause discomfort or stress to the animals (blood samplings) were performed under mild ketamine-xylazine sedation i.m. The use and treatment of the rabbits was in accordance with the European Communities Council Directive of September 22, 2010 (276/33/20.10.2010) and the protocol was approved by the competent Veterinary Directorate of Athens Prefecture (Approval No.: 4141/3.11.2011).

Animals were allowed 2 weeks of acclimatization after arrival and then were randomly separated in three experimental groups. Animals in the Control group (*n* = 6) were fed standard commercial rabbit diet (ND), animals in the Cholesterol group (*n* = 7) were fed the standard diet enriched with 1% *w*/w cholesterol (CD) while animals in the Cholesterol + Colchicine group (*n* = 10) were fed the same cholesterol-enriched diet plus 2 mg/kg body weight/day colchicine (CD + Col)*.* Standard rabbit chow [Conigli Svezzamento, S.I.V.A.M. Società Italiana Veterinaria Agricola Milano S.P.A., Casalpusterlengo (LO), Italy] consisted of the following (w/w): 37% carbohydrates, 16% proteins, 4% fat, 15% fiber, 11% water, 8% ash and an appropriate mixture of minerals and vitamins for the healthy subsistence of the animals in the laboratory (added to the premix by the manufacturer). The atherogenic food was prepared by dissolving the appropriate amount of cholesterol in diethyl ether (without butylated hydroxytoluene as inhibitor) and adding the mixture to the rabbit chow. After ether evaporation, cholesterol food was kept at −20 °C until use. Following the practice of the 3Rs (Replacement, Refinement, Reduction), we chose to separate the animals unequally in those 3 experimental groups. The ND group underwent the least of the interventions and we decided to maintain the smallest number of animals as possible. On the other hand, the results of CD group are anticipated to a greater extent, as shown in previous researches [24]. For that reason high concentration of 1% *w*/w cholesterol was added in the standard rabbit chow expecting greater atherosclerotic lesions. The feeding duration was 7 weeks, as proposed by other researchers [21]. Colchicine was pulverized and was then dissolved in tap water. The 3 groups of animals had free access to their food and tap water throughout the experiment. CD + Col group’s daily intake of colchicine through tap water was 2 mg/kg of body weight. We ensured it by measuring water consumption daily and adjusting colchicine dilution. High but not lethal dose of 2 mg/kg/d was selected since there is evidence that per os low dose colchicine did not affect the progression of atherosclerotic plaques [29]. We decided to administer colchicine through drinking water in order to minimize the stress caused to the animals due to the injections that could affect the measured serum parameters related to inflammation.

Blood samples were collected at 0(T0), 4(T1) and 7(T2) weeks of dietary intervention from the central ear artery after overnight fasting. The animals were euthanized by administration of an intravenous overdose of sodium pentobarbital preceded by i.m. ketamine-xylazine sedation at 7 weeks. Their aortas were removed from the arch to the iliac bifurcation and the perivascular adipose tissue was excised.

### Blood analyses

#### Biochemical measurements

Blood samples were collected for the determination of total cholesterol (TC), HDL cholesterol (HDL-C), LDL cholesterol (LDL-C), triglycerides (TG), glucose (Glu), creatinine (Cr), total protein (TP), γ GT, alkaline phosphatase (ALP), aspartate aminotransferase (AST) and alanine aminotransferase (ALT) levels. Samples were stored at −80 °C until analysis.

#### ELISA

Serum leptin, IL-18 and insulin levels were determined by enzyme-linked immunosorbent assay (ELISA) using commercially available kits. The optical density (OD value) of each well was determined, using a micro-plate reader (BIORAD 630), to 450 nm. (Rabbit Insulin Elisa kit, Elabscience Biotechnology Co., Ltd., Catalog No E-EL-RB2274, www.elabscience.com - Rabbit Interleukin 18(IL-18) Elisa kit, Biomatik USA, LLC, Catalog No EKU05257, www.biomatik.com and Serum leptin elisa kit for rabbit, Biomatik USA, LLC, Catalog No EKU 05597, www.biomatik.com).

#### Tissue analysis

##### Histology

Histologic examination of the aorta was performed as described from Yanni et al. [21]. In brief, the isolated aorta was cleaned from the connective and adipose tissue and was cut open longitudinally. The vessel was fixed to retain initial dimensions and then was embedded in a neutral 10% (*v*/v) buffered formalin solution for 24 h. Representative parts of the ascending, thoracic and abdominal aorta were excised and processed for paraffin incubation. Paraffin sections of 3 μm thickness of aortas were cut and stained with eosin and hematoxylin for further microscopic examination. An expert blinded to the intervention groups performed the histologic examination.

##### Image analysis

The evaluation of the aortic intimal thickness was performed using image analysis [21]. Images were digitalized using a light microscope Eclipse 80i (Nikon Corp., Tokyo, Japan) with an attached digital camera (DS-2 MW, Nikon Corp.). Images were loaded to a computer equipped with the appropriate software (Image ProPlus v 5.1, MediaCybernetics, MD, USA) and the layers of the aorta (adventitia, media, intima) were measured. The thickness of the intima was calculated automatically.

#### Statistical analysis

For the statistical analysis of these data, we chose to work with non-parametric tests, because of the rather small sample size and the non-normality of the distributions of interest. The statistical analysis included: a) paired-sample Wilcoxon test, b) Spearman correlation coefficient and c) Mann-Whitney U Test. Results were expressed as the mean ± SD. In all cases where multiple comparisons have been performed, Benjamini-Hochberg’s False Discovery Rate has been utilized in order to assess pairwise significance levels, while controlling family-wise type I errors. Statistical analysis was performed in IBM SPSS v23 (IBM Corp. Released 2015. IBM SPSS Statistics for Windows, Version 23.0. Armonk, NY: IBM Corp.). A value of *p* < 0.05 was considered statistically significant.

## Results

### Effect of colchicine on growth and body weight

During the experiment, all of the rabbits gained weight and no statistical difference was observed between groups receiving the various dietary interventions. The body weight of the animals in the three groups at baseline, four and 7 weeks of the study is presented in Table 1.

**Table 1:** Body weight, Glucose, plasma lipids, Liver Function Tests, Creatinine and Total Protein in the three groups of animals during the entire experimental period^α^

	Group	0 weeks	4 weeks	7 weeks
Body weight (kg)	ND	3.5 ± 0.2	3.7 ± 0.2^a^	3.9 ± 0.2^a,b^
	CD	3.6 ± 0.2	3.8 ± 0.2^a^	3.9 ± 0.1^a^
	CD + Col	3.5 ± 0.1	3.7 ± 0.2^a^	3.8 ± 0.1^a,b^
Glu (mg/dL)	ND	165.00 ± 36.17	136.00 ± 25.62	164.17 ± 17.18
	CD	159.86 ± 15.76	195.00 ± 41.05^c,e^	198.29 ± 27.53^e^
	CD + Col	170.20 ± 26.54	143.50 ± 25.78	157.10 ± 30.98
TC (mg/dL)	ND	56.33 ± 12.14	40.33 ± 13.06	35.83 ± 8.42
	CD	51.86 ± 12.25	404.43 ± 36.97^a,c^	449.86 ± 8.78^a,b,c^
	CD + Col	54.70 ± 18.01	430.90 ± 25.74^a,c^	453.90 ± 12.33^a,b,c^
HDL-C (mg/dL)	ND	17.67 ± 1.63	28.17 ± 7.57	27.33 ± 6.74
	CD	16.14 ± 2.67	81.71 ± 18.54^a,c^	117.14 ± 22.34^a,b,c^
	CD + Col	16.50 ± 4.48	127.00 ± 28.78^a,c,d^	109.10 ± 37.10^a,b,c^
LDL-C (mg/dL)	ND	19.52 ± 6.62	3.33 ± 6.62^a,d,e^	2.33 ± 2.42^a^
	CD	17.28 ± 6.51	315.57 ± 23.64^a,c^	306.57 ± 28.86^a,c^
	CD + Col	19.89 ± 12.42	294.78 ± 26.71^a,c^	334.50 ± 35.26^a,b,c^
TG (mg/dL)	ND	99.83 ± 42.68	47.00 ± 13.86^a^	31.17 ± 4.40^a,b,d^
	CD	91.00 ± 35.75	34.14 ± 15.54^a^	130.00 ± 133.18^b^
	CD + Col	90.20 ± 31.31	42.80 ± 17.40^a^	50.60 ± 32.19^a,b^
SGOT/AST (U/L)	ND	20.42 ± 5.42	30.67 ± 6.83^a^	29.83 ± 4.02^a^
	CD	19.92 ± 6.08	39.14 ± 9.67^a,e^	22.50 ± 4.04^b,c,e^
	CD + Col	21.69 ± 7.48	25.80 ± 8.61	41.70 ± 20.71
SGPT/ALT (U/L)	ND	28.81 ± 9.57	49.17 ± 11.55^a^	41.33 ± 10.80^a^
	CD	33.14 ± 10.10	41.86 ± 10.54	30.00 ± 13.51
	CD + Col	35.35 ± 11.84	47.70 ± 16.20	56.70 ± 29.93
γGT (U/L)	ND	9.19 ± 1.97	13.17 ± 3.19	11.17 ± 1.86
	CD	9.56 ± 0.93	11.29 ± 2.69	20.00 ± 23.93
	CD + Col	9.65 ± 1.29	11.70 ± 2.83^a^	9.30 ± 2.21^a,b^
ALP (U/L)	ND	66.67 ± 23.31	105.23 ± 31.63	78.77 ± 20.85
	CD	69.00 ± 6.83	95.40 ± 27.07	54.73 ± 40.37
	CD + Col	73.10 ± 14.77	107.87 ± 11.75^a^	52.67 ± 18.29^a,b^
Creatinine (mg/dL)	ND	1.08 ± 0.6	1.18 ± 0.07	1.07 ± 0.08
	CD	1.12 ± 0.16	1.24 ± 0.10	1.18 ± 0.13
	CD + Col	1.08 ± 0.17	1.05 ± 0.14^c,d^	1.13 ± 0.08
Total protein (g/dL)	ND	5.75 ± 0.24	6.20 ± 0.38	5.82 ± 0.29
	CD	5.88 ± 0.23	6.47 ± 0.26	6.28 ± 0.86
	CD + Col	5.73 ± 0.34	6.09 ± 0.47	5.96 ± 0.26

Paired samples Wilcoxon test adjusted with Benjamini-Hochberg procedure

^α^Data are presented as mean ± SD, ^a^ *p* < 0.05 vs. baseline, ^b^ *p* < 0.05 vs. 4 weeks, ^c^ *p* < 0.05 vs. ND group, ^d^ *p* < 0.05 vs. CD group, ^e^ *p* < 0.05 vs. CD + Col group – ND (*n* = 6), CD (*n* = 7), CD + Col (*n* = 10)

### Biochemical measurements

As presented in Table 1, the baseline laboratory measurements of all groups were comparable at the beginning of the experiment.

**Fig. 1:**
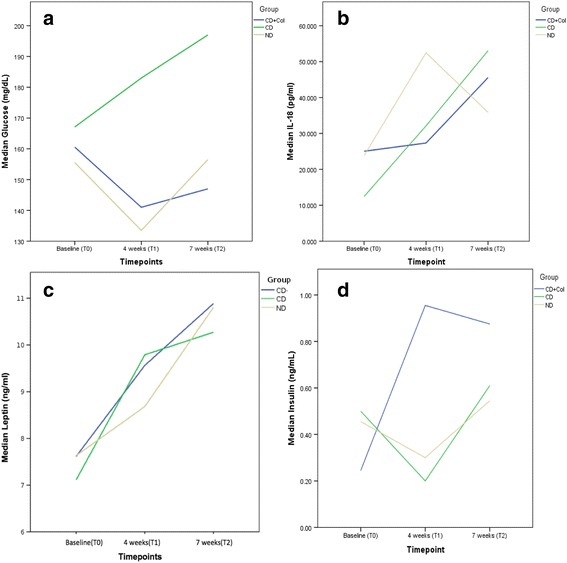
Measure of Glucose, IL-18, Leptin and Insulin levels. **a** The trend of median glucose levels during the experiment for ND, CD and CD + Col groups. **b** Median IL-18 values of the 3 groups during follow up. **c** Median Leptin values of groups during follow up. **d** Median insulin values of the 3 groups during follow up

Plasma glucose levels were statistically significantly increased in CD group in the 4th and 7th week of the experiment in comparison with the CD + Col (*p* < 0.05 in 4 weeks and 7 weeks for CD + Col group) and only in the 4th week in comparison with the ND group (*p* < 0.05) Fig. 1a.

Plasma Total Cholesterol and LDL-C were significantly elevated in CD and CD + Col groups in comparison with the ND group during the experiment (*p* < 0.05), but without statistical difference between these 2 groups.

HDL-C was significantly increased in CD + Col group in comparison with CD group in the 4th week of the study (*p* < 0.05) but this difference was not observed in the 7th week (*p* > 0.05). In particular, the levels of HDL-C in CD + Col in the 7th week were lower than those in the 4th week of the experiment. These 2 groups had significantly raised levels of HDL-C in comparison with the ND group during the 7 weeks of the intervention (*p* < 0.05).

TG levels decreased for all groups (*p* < 0.05 compared to the baseline for each group) at the 4th week but the increase in CD group was statistically significant in comparison with the CD + Col group between 4th and 7th week (*p* < 0.05). In the end of the experiment CD + Col group had statistically significant lower TG levels (*p* < 0.05) than in the beginning, whereas CD group had increased TG levels but not statistically significant compared to the beginning.

Regarding the liver enzymes, SGOT/AST levels were significantly increased in the CD group in the 4th week in comparison with the CD + Col group (*p* < 0.05) but were significantly decreased in the end of the experiment (*p* < 0.005 vs. CD + Col, *p* < 0.05 vs. ND and *p* < 0.05 for the CD group between 4th and 7th week). SGPT/ALT levels showed an increasing trend for all groups in the middle of the experiment (*p* < 0.05 for ND group) but decreased at the end (vs.baseline), apart from the CD + Col group that preserved the non statistically significant increase. Finally, γ GT levels were significantly increased in CD + Col at the 4th week (*p* < 0.05 vs.baseline) but decreased at the 7th week (*p* < 0.05 vs.baseline, *p* < 0.05 vs 4th week), however there was no significant difference between groups (*p* > 0.05 within the period of the experiment). ALP was significantly increased only in the CD + Col group in the middle of the experiment (*p* < 0.05 vs. baseline) but decreased at the end of it (*p* < 0.05 vs. 4th week, *p* < 0.05 vs. baseline). There was no significant difference between groups, though.

Creatinine levels were significantly lower at the 4th week of the experiment in the CD + Col group (*p* < 0.05 vs. ND and *p* < 0.01 vs. CD) but this significance was not retained to the 7th week.

### Effect of colchicine on the levels of IL-18, Leptin and Insulin

The measured levels of IL-18, Insulin and Leptin are presented in Table 2 *.*

**Table 2:** Levels of IL-18, Leptin and Insulin in the three groups of animals during the entire experimental period^α^

	Group	0 weeks	4 weeks	7 weeks
IL-18 (pg/mL)	ND	37.23 ± 31.87	80.49 ± 88.48	74.30 ± 104.17
	CD	18.93 ± 14.29	45.35 ± 33.46^a^	42.72 ± 28.73^a^
	CD + Col	52.37 ± 46.17	31.23 ± 20.09	38.49 ± 23.57
Leptin (ng/mL)	ND	7.63 ± 4.79	8.68 ± 3.50	10.80 ± 1.16
	CD	7.12 ± 5.54	9.79 ± 0.48	10.27 ± 0.79^b^
	CD + Col	7.61 ± 4.47	9.56 ± 0.95	10.89 ± 0.68^a,b^
Insulin (ng/mL)	ND	0.48 ± 0.26	0.34 ± 0.08	0.63 ± 0.2^a,b^
	CD	0.55 ± 0.39	0.20 ± 0.04^a^	0.89 ± 0.4^a,b^
	CD + Col	0.44 ± 0.33	0.86 ± 0.30^a,c,d^	0.86 ± 0.29^a^

Paired samples Wilcoxon test adjusted with Benjamini-Hochberg procedure

^α^Data are presented as mean ± SD, ^a^ *p* < 0.05 vs. baseline, ^b^ *p* < 0.05 vs. 4 weeks, ^c^ *p* < 0.05 vs. ND group, ^d^ *p* < 0.05 vs. CD group – ND (*n* = 6), CD (*n* = 7), CD + Col (*n* = 10)

IL-18 levels increased in the ND group but without statistical difference (vs baseline, *p* > 0.05). In CD group, IL-18 levels were significantly increased at the 4th week of the experiment vs baseline (*p* < 0.05) and this significance was noticed also at the 7th week of the experiment (*p* < 0.05 vs baseline). There was not any statistical difference between groups Fig. 1b.

Leptin levels were gradually increased during the experiment for all groups. This increase in CD + Col group was significant only between 4th and 7th week and at the 7th week vs baseline (*p* < 0.05). We noticed also a statistically significant increase in the CD group between 4th and 7th week (*p* < 0.05). No statistical difference was observed between groups Fig. 1c.

A mean two-fold increase in insulin levels was observed in CD + Col group at the 4th and 7th week of the study (*p* < 0.05 vs baseline) but there was no difference between 4th and 7th week (*p* > 0.05). In CD group insulin levels were increased throughout the experiment (*p* < 0.05, 4th week vs baseline, 7th vs 4th week, 7th week vs baseline), while in ND group statistically significant increase was noticed between 7th and 4th week and 7th week vs baseline (*p* < 0.05). The levels of insulin in CD + Col group were significantly higher in comparison with the other 2 groups (*p* < 0.05) in the 4th week. The same applies to CD vs ND group (*p* < 0.05) Fig. 1d.

### Correlations between IL-18, Leptin, Insulin and other measured parameters

We performed Spearman’s correlation between IL-18, leptin and intima thickness to investigate whether these variables were linearly correlated. There was not any statistically significant correlation between the levels of IL-18 and the intima thickness. The same applies to the correlation between leptin and intima thickness.

The same test was used to determine the possible relationship between IL-18 and insulin and plasma glucose levels. In CD + Col group there was a strong, positive linear correlation between IL-18 and insulin levels in the 4th week (*r*_s_ = .66, *n* = 10, *p* < 0.05), whereas in the 7th week this correlation became strong negative (*r*_s_ = −.86, *n* = 10, *p* < 0.05). Apart from the aforementioned positive relationship, all Spearman’s correlations were negative between the measured values. Statistical significance was also found in the 4th week in the ND group (*r*_s_ = −.94, *n* = 6, *p* < 0.05). Despite the fact that some correlations between insulin and IL-18 were found, IL-18 levels did not show any correlation with glucose.

We also conducted Spearman correlation coefficient to determine the relationship between weight and leptin. The results were not statistically significant in any group in any week of measurement (data not shown). For week 7, the results for CD + Col group were marginally not significant (*r*_s_ = −.0.63, *n* = 10, *p* = 0.051).

### Histology

**Fig. 2:**
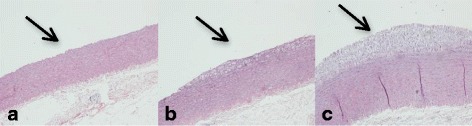
Representative thoracic aortic sections of rabbit groups **a** ND, **b** CD, **c** CD + Col. Hematoxylin–eosin stain, 100× magnification. Atherosclerotic lesions abundant in foam cells

Intima thickness was measured in parts of the ascending, thoracic and abdominal aorta and the results are represented in Table 3 and Fig. 2 *.* We observed fatty streaks only in the CD and the CD + Col group, whereas the ND group did not develop atherosclerotic lesions. Intima thickness in the ascending aorta of the CD + Col group was significantly greater than that of CD and ND groups (*p* < 0.05). The same statistically significant difference between groups was observed in the thoracic aorta (*p* < 0.05), but there was not any difference in the abdominal aorta.

**Table 3:** Intima Thickness of Different Sections of Aorta^α^

Group	ND (*n* = 6)	CD (*n* = 7)	CD + Col (*n* = 10)
Ascending aorta	4.20 ± 0.98	16.7 ± 12.49	35.67 ± 28.58^a^
Thoracic aorta	7.97 ± 4.26	14.39 ± 14.21	23.59 ± 14.77^a^
Abdominal aorta	6.42 ± 5.63	19.89 ± 19.99	21.56 ± 17.55

Paired samples Wilcoxon test adjusted with Benjamini-Hochberg procedure

^α^Values are expressed in μm, mean score ± SD, ^a^ *p* < 0.05 vs. ND group

## Discussion

In the present study we intended to investigate whether per os administration of colchicine in cholesterol-fed rabbits inhibits de novo the progression of atherosclerotic lesions and if it suppresses the inflammation that accompanies atherosclerosis. Our notable findings are that colchicine does not affect the development of atherosclerotic plaques. On the contrary, per os colchicine seems to favor the creation of fatty streaks in the aorta of rabbits. Moreover, serum levels of IL-18, leptin and insulin were not affected by the administration of colchicine.

The role of inflammation in the development of atherosclerosis is of outmost importance [30, 31]. Considering the well-known anti-inflammatory properties of colchicine, we selected a high cholesterol diet induced animal model that is characterized by early development of atherosclerotic lesions following the intervention as well as inflammation [32].

Previous studies have shown diverse results concerning the effect of colchicine on the evolution of atherosclerotic disease. Hollander W. et al. [33] was the first to point out the role of colchicine in the inhibition of the formation of atherosclerotic lesions in rabbits. The results of the study of Wójcicki et al. [34], who injected *ip* colchicine in cholesterol-fed rabbits, were in accordance with the aforementioned findings. Colchicine, though, did not have the same effect either in the same animal model with pre-established atherosclerotic lesions [29], or in pigs [35]. Intimal hyperplasia after ballooning or stent placement in atherosclerotic vessels, which uses a similar mechanism of smooth muscle cells migration and proliferation as in atherogenesis [36], was studied intensively. Experiments in animal models [37–39] and in humans [40, 41] had different results regarding concurrent administration of colchicine and stent placement or just balloon angioplasty. In our study, we used the rabbit animal model, as it is known that the consumption of cholesterol enriched food leads to the creation of atherosclerotic lesions that resemble the early stages of atherosclerosis in humans. The administration of 1% (*w*/w) cholesterol diet for 7 weeks created the requested model as can be confirmed by the elevation of plasma cholesterol and the characteristic atherosclerotic lesions of the aorta, Fig. 1. Interestingly, oral administration of colchicine 2 mg/kg/d, considered a high dose compared with the aforementioned studies in animals, did not reduce intimal hyperplasia in the CD + Col group in comparison with the CD group, as we would expect. Especially, early atherosclerotic lesions in the CD + Col group were significantly greater in the ascending and thoracic aorta compared to the other 2 groups. This may be explained by the fact that a different route of administration for colchicine was chosen. Although no diarrheas or loss of weight was observed, the absorption of colchicine from the gastrointestinal tract and the subsequent bioavailability may be hindered. Another explanation could be that colchicine affects later stages of formation of atherosclerotic plaques. Further studies with longer treatment periods are needed to elucidate in which stage colchicine affects the progression of atherosclerosis and what dosage should be administered.

In the present study we observed that colchicine confirmed its role as a hypoglycemic drug lowering plasma glucose levels [42, 43], as can be noticed in the CD + Col group. Concerning the lipid profile of the rabbits, we observed that cholesterol enriched food increased total plasma cholesterol and LDL-C in both treatment groups, whereas TG levels were reduced in the end of the experiment in a statistically significant manner for the CD + Col group. Despite the fact that the atherogenic role of TGs is known [44–46], the way colchicine reduces TG levels has not yet been described. Colchicine could act as niacin and increase apoliprotein B (apoB) degradation leading to reduced formation of VLDL [47] or as an antagonist of the bile acid receptor farnesoid X receptor (FXR) resembling the lipid-lowering activity of guggulsterone [48]. Eicosapentaenoic acid ethyl ester is reported to lower TG levels by reducing apolipoprotein B and lipoprotein-associated phospholipase A_2_ levels [49]. Moreover, colchicine could inhibit acyl CoA/diacylglycerol acyltransferase (DGAT) 1, as King et al. showed in their study using a small-molecule inhibitor of DGAT 1 [50]. Further studies are needed to clarify the mechanism of colchicine’s action on the metabolism of TG with possible use of radiolabeled acetate or glycerol as described by Jin et al. or measurement of apoB levels.

HDL-C levels were significantly higher in the CD + Col group in the middle of the experimental period, but without retaining this statistical difference until the end. High levels of HDL-C are known to be atheroprotective, thus its decrease in the 7th week could be further explored in other studies by sacrificing animals in the 4th week of the intervention and evaluating the atherosclerotic lesions. As for the serum liver enzymes, SGOT/AST followed the course of HDL-C, while SGPT/ALT, γ GΤ and ALP showed no significant differences between the groups, indicating a non-harmful role of colchicine on liver function. Of note, creatinine levels of the CD + Col group in the middle of the experiment were significantly lower compared with the other 2 groups, but this difference was not observed in the end.

To the best of our knowledge, this is the first study to correlate serum IL-18 levels with the progression of atherosclerotic disease in cholesterol-fed rabbits that were treated with colchicine. IL-18, as a member of IL-1 family, is regulated by the activation of NLRP3 inflammasome [7] and has been proved to play a pro-inflammatory role in the pathogenesis of atherosclerosis. Raised serum levels of IL-18 have been associated with increased intima thickness [51, 52] and plaque instability [9, 53], while administration of IL-18 binding protein in *apoE* ^*−/−*^ mice reduced the development of atherosclerotic lesions [54]. Colchicine is used as an anti-inflammatory drug in diseases like gout, pericarditis and familial Mediterranean fever. Colchicine inhibits the activation of the inflammasome NLRP3 in neutrophils by binding to microtubules that are involved in the spatial arrangement of mitochondria [19] and, therefore, reduces production of inflammatory cytokines [55]. Recently, several researchers tried to associate the effect of colchicine on the levels of IL-18 and the stability of atherosclerotic plaques. Martinez et al. [18] suggested that short-term colchicine treatment reduces local production of IL-18 in ACS patients, but the same treatment did not have the same efficacy on isolated monocytes in peripheral blood [56]. The fact that colchicine prevents plaques from rupture is supported by the LoDoCo trial [17]. Apparently, the acute events that take place during the rupture of an atherosclerotic plaque are different from the de novo formation of it. This can explain the fact that in our study IL-18 levels of all groups did not differ statistically, indicating a low-grade inflammation process during the experiment. Studies that were focused on the NLRP3 pathway activation and its direct role on atherosclerosis showed controversial results [4, 57]. This indicates a mechanism in atherosclerosis that does not implicate necessarily participation of the NLRP3 inflammasome. In our experiment, we found neither a possible effect of colchicine on serum IL-18 nor an association between IL-18 and intima thickness, except for a suggestive negative correlation between IL-18 and intima thickness of the thoracic aorta in the CD + Col group. This may be indicative of a different mechanism of inflammation in the progression of atherosclerotic disease. Mechanisms that either upregulate the levels of the NLRP3 inflammasome or protect it against the destabilizing role of colchicine may be developed. The fact that there is a trend towards an increase in intima thickness in the CD + Col group in thoracic aorta specimens, although low IL-18 levels were measured, proposes that the inflammasome pathway was bypassed or low-grade inflammatory conditions, as the one that takes place during de novo formation of atherosclerotic plaques, may not be mirrored adequately by serum levels of IL-18. The PRIME study showed that IL-18 may serve as a biomarker for future coronary events in healthy subjects [58]. However, the study population included men aged 50–59 years; namely humans with pre-established atherosclerotic lesions to a certain degree. Additional studies with a larger number of animals will be needed to determine the relationship between colchicine administration, serum and local IL-18 levels and intima thickness.

IL-18 has also been implicated in the evolution of diabetes. Lewis et al. [12] proposed that IL-18 induced macrophages in transplanted pancreatic islets to produce chemokines which caused islet graft failure, while Jourdan et al. [11] showed similar results in ZDF rats. An other study showed that in NOD mice, infiltrated macrophages in pancreatic cells expressed high levels of IL-18, which is considered to cause insulitis, a preclinical state of diabetes [13]. Studies in humans that either had a high risk of developing type 1 and 2 diabetes (T1D, T2D) [59, 60] or were already diagnosed with these diseases [61–66], revealed elevated serum levels of IL-18. In addition, some investigators correlated high levels of IL-18 and insulin resistance in patients with T2D, obesity or metabolic syndrome [67–71]. In our experiment, there was a significant increase in insulin in the CD + Col group in the 4th week and 7th week vs baseline and its insulin levels remained high and comparable to those of the CD group until the end. Despite the fact that we did not notice any impact of colchicine on serum IL-18 levels, it seems that colchicine had a greater inhibitory effect on the developed inflammation in pancreas that destroys β-cells. In the CD + Col group we noticed a strong positive correlation between IL-18 and insulin levels in the 4th week that was inversed to a strong negative correlation in the 7th week. This may be explained by the fact that colchicine inhibits the NLRP3 inflammasome in pancreas partially. The progression of the inflammation may be hindered in the middle of the experiment but eventually local pancreatic inflammation induced a greater beta cell apoptosis and lower insulin levels. Notably, the body weight of the rabbits remained comparable throughout the experiment, showing that obesity-induced insulin resistance was probably not associated with the levels of insulin. In this study, we did not measure local pancreatic IL-18 levels in order to ascertain the possible suppression or exaggeration of local inflammation. In spite of this result there was not any correlation between IL-18 and glucose, which probably means that reduced glucose levels in the CD + Col group are regulated by other mechanisms such as impaired hepatic gluconeogenesis and/or attenuated glycogenolysis. Further studies are required, where pancreatic specimens will be examined, in order to define macrophage infiltration and local levels of IL-18.

Another fact that concerned our study is the possible effect that colchicine had on the levels of leptin and body weight and the role of leptin in the development of atherosclerotic lesions. It is known that leptin is secreted mainly by adipose tissue and its levels are in direct relationship with its mass [72]. Results from various studies that examine the effect of leptin on atherosclerosis are contradictory. Researchers used various experimental models that presented either the atherogenic [14, 15, 73–75] or the anti-atherogenic role [76–79] of leptin. To our knowledge, this is the first study to examine in vivo the way colchicine affects leptin levels and examines leptin’s possible impact on atherosclerosis. In our experiment, we did not find any statistical difference between groups concerning the levels of leptin during the experiment. Despite the fact that body weights of the rabbits were not significantly different among the groups, a marginally not significant negative correlation of leptin and body weight was found in the CD + Col group (*p* = 0.051) in the 7th week, showing a stronger negative relationship between body weight and leptin levels than in the CD group (*r*_s_ = −.58, *n* = 7, *p* > 0.1). Further experiments are required, though, to correlate the total amount of adipose tissue and colchicine administration. Concerning the role of leptin in atherosclerosis, we did not deduce a clear role of it. Colchicine did not have a deterrent effect on the development of atherosclerosis either directly or indirectly by regulating the levels of leptin. Nevertheless, a suggestive negative correlation between the CD group and intima thickness in ascending aorta specimens, potentially proposes an antiatherogenic role of leptin.

Our study has several limitations. First, we distributed rabbits in 3 groups unequally. The reasons for this separation are explained above. Second, we used only one group of a single dose of colchicine, trying to examine atherosclerosis progress through a high but not lethal dose of colchicine. The decision was taken examining the results of relevant studies that used low colchicine dose [29, 35]. Third, the primary end point was measurement of biochemical parameters, aortic intima thickness and serum levels of IL-18, insulin and leptin at the end of the experiment. The present study was not designed to measure local expression of IL-18 (pancreatic and vascular), the amount of leptin receptors and macrophage infiltration of pancreas or apolipoprotein B levels. Molecular-based experiments have to be designed, examining the possible local effects of colchicine on vessels and different tissues, like pancreas and liver. In that way, light will be shed on mechanisms with which colchicine attenuates local inflammation and triglyceride levels.

## Conclusions

In summary, our results suggest that per os administration of colchicine did not influence atherosclerosis progression in cholesterol-fed rabbits. We encountered unexpected findings like the possible attenuating role of colchicine on TG levels. Levels of IL-18, insulin and leptin did not differ statistically between groups. Further research is warranted regarding the effect of colchicine on the NLRP3 inflammasome in vitro and in vivo and its role in the pathogenesis of atherosclerosis and pancreatic inflammation.

## Abbreviations

ACS: Acute coronary syndrome
ALP: Alkaline phosphatase
ALT: Alanine aminotransferase
ApoB: Apolipoprotein B
AST: Aspartate aminotransferase
CD + Col: Cholesterol diet plus colchicine 2 mg/kg body weight/day
CD: Cholesterol diet 1% *w*/w
Cr: Creatinine
Glu: Glucose
HDL-C: HDL cholesterol
IL-1β and IL-18: Interleukin-1β and -18
LDL-C: LDL cholesterol
LDLR: Low-density lipoprotein receptor
ND: Standard rabbit diet
NLRP 3: NOD-like receptor protein 3
NOD: Non-obese diabetic
T1D: Type 1 diabetes
T2D: Type 2 diabetes
TC: Total cholesterol
TG: Triglycerides
TP: Total protein
